# Dexamethasone is non-inferior to antihistamine plus dexamethasone premedication in preventing ramucirumab plus nab-paclitaxel infusion-related reactions in gastric cancer: a multicenter retrospective study

**DOI:** 10.1007/s00520-024-08910-8

**Published:** 2024-10-07

**Authors:** Yutaka Negoro, Taichi Maeda, Hiroyuki Igarashi, Mina Shigemori, Toshihiro Tanaka, Yukio Ito, Norihiko Tanizawa, Shota Nishikawa, Jyunya Ogawa, Yukio Kamitani, Kyohei Watanabe, Hitoshi Tsukamoto, Nobuyuki Goto

**Affiliations:** 1 Department of Pharmacy, University of Fukui Hospital, Yoshida-Gun, 23-3 Matsuoka-Shimoaizuki, Eiheiji-Cho, Fukui, Japan; 2 Department of Pharmacy, Japanese Red Cross Fukui Hospital, Fukui, Japan; 3 Department of Pharmacy, Fukui-Ken Saiseikai Hospital, Fukui, Japan; 4 Medical Research Support Center, University of Fukui Hospital, Yoshida-Gun, Fukui, Japan

**Keywords:** Ramucirumab, Infusion-related reactions, Antihistamines premedication, Nab-Paclitaxel, Gastric cancer

## Abstract

**Purpose:**

Ramucirumab (RAM) is recommended as premedication with H_1_-receptor antagonists (H_1_RA) to prevent infusion-related reactions (IRRs). However, RAM is a human antibody with a low incidence of IRRs. We evaluated the noninferiority of non-H_1_RA (dexamethasone [DEX] alone) premedication to H_1_RA (plus DEX) premedication in terms of IRRs in patients with gastric cancer receiving RAM plus nanoparticle albumin-bound paclitaxel (nab-PTX).

**Methods:**

This was a noninferiority, multicenter, retrospective trial conducted in three Japanese centers to assess the incidence of IRRs in patients receiving RAM plus nab-PTX for gastric cancer between 2018 and 2023. Patients with gastric cancer receiving RAM plus nab-PTX were divided into groups with and without H_1_RA premedication. The incidence of IRRs was compared between the two groups.

**Results:**

Ninety patients were evaluated, with non-H_1_RA and H_1_RA premedications in 43 and 47 cases, respectively. After the first dose of RAM, IRRs were not observed in either group. IRRs during the overall doses were 0% for non-H_1_RA premedication and 2.1% for H_1_RA premedication (90% confidence interval (CI): –5.6%–1.3% for each comparison). The upper limit of the 90% CI (1.3%) did not exceed the noninferiority margin (Δ) of + 10% and therefore met the noninferiority criteria.

**Conclusion:**

RAM plus nab-PTX for gastric cancer with DEX premedication may be possible without H_1_RA premedication.

**Supplementary Information:**

The online version contains supplementary material available at 10.1007/s00520-024-08910-8.

## Introduction

Ramucirumab (RAM), a human anti-vascular endothelial growth factor receptor-2 monoclonal antibody, is a key chemotherapeutic drug for gastric, colorectal, non-small cell lung, and hepatocellular cancers [1–4]. It is used as a second-line treatment in combination with paclitaxel (PTX) for advanced or recurrent gastric cancer [5, 6].

Infusion-related reactions (IRRs) are well-known adverse events associated with drugs [7]. In general, practices to reduce the IRR risk of monoclonal antibody drugs include premedication with antihistamines and/or corticosteroids and reducing the rate of infusion [8]. When RAM is administered, premedication with H_1_-receptor antagonists (H_1_RA) is recommended to prevent IRRs. PTX requires premedication with H_1_RA, H_2_-receptor antagonists, and corticosteroids prior to administration because of the high incidence of hypersensitivity [9]. Conversely, nanoparticle albumin-bound paclitaxel (nab-PTX) is a formulation developed without the use of solvents that cause hypersensitivity to PTX [10]. Therefore, nab-PTX is associated with a lower risk of hypersensitivity than PTX, and in general, premedication is not required. A phase 3 trial in advanced gastric cancer demonstrated the noninferiority of nab-PTX to PTX in terms of overall survival [11]. In addition, RAM plus nab-PTX has shown efficacy and safety similar to those of RAM plus PTX in the secondary treatment of advanced gastric cancer [12–14]. In the RAM plus nab-PTX, H_1_RA is commonly administered to prevent RAM-induced IRRs, although nab-PTX does not require premedication for hypersensitivity. However, the anticholinergic effects of H_1_RA limit its use in patients with angle-closure glaucoma or benign prostatic hyperplasia. Furthermore, the drowsiness and dizziness caused by H_1_RA compromise driving [15].

RAM plus nab-PTX has a low emetic risk, and dexamethasone (DEX) is often used as premedication according to the antiemetic guidelines [16–18]. DEX may be premedicated with monoclonal antibody drugs to prevent IRR development [8]. A previous observational study reported that RAM-induced IRRs without H_1_RA premedication were low in patients with solid cancers receiving various RAM-containing regimens [19]. In these reports, many patients treated with RAM were premedicated with DEX. However, in gastric cancer patients receiving RAM plus nab-PTX, the effect of DEX alone, without H_1_RA, on IRRs remains unclear. If RAM plus nab-PTX could be administered without the need for H_1_RA, this approach would offer advantages over RAM plus PTX, which requires H_1_RA premedication. In this study, we investigated the noninferiority of DEX without H_1_RA to DEX with H_1_RA in preventing IRRs in patients with gastric cancer receiving RAM plus nab-PTX.

## Materials and methods

We conducted a retrospective review of the medical records of gastric cancer patients receiving RAM plus nab-PTX between March 1, 2018, and March 31, 2023, at three hospitals in Japan. Patients received RAM intravenously at least every 14 days, with an initial RAM infusion for 60 min and subsequent infusions for 60 or 30 min. Patients were excluded if they (1) were previously treated with RAM, (2) received daily H_1_RA or steroids, or (3) were not premedicated with DEX. The patients were categorized into two groups based on the use of H_1_RA premedication. The primary endpoint was the incidence of IRRs during the initial RAM infusion between the two groups. The secondary endpoints were the incidence of IRRs during the total number of doses and infusion durations (30 and 60 min) between the two groups. The incidence of suspected drowsiness due to premedication was also assessed. IRR severity was assessed according to the National Cancer Institute Common Terminology Criteria for Adverse Events, version 5.0. IRRs were defined as grade ≥ 1.

This study was approved by the Research Ethics Review Committee for Medical Research at the University of Fukui (Reference No. 20220219). The patients were informed on the hospital website that they could opt out of the study at any time.

### Statistical analysis

We analyzed the differences in patient background factors between the two groups using the Mann–Whitney *U* test for continuous variables and Fisher's exact test for categorical variables. In the RAINBOW study, which mandated premedication with H_1_RA and DEX, the incidence of IRRs was 5.8% among patients receiving RAM plus PTX for gastric cancer [1]. Accordingly, the sample size for this study assumed a RAM-induced IRR incidence of approximately 5.8% with premedication using H_1_RA and DEX. To date, there are no studies comparing the effectiveness of various premedications in preventing RAM-induced IRRs. Thus, we set a noninferiority margin for the prevention of IRRs based on the findings of the MABEL study [20], which evaluated the efficacy of combining H_1_RA and DEX as premedication against H_1_RA alone during cetuximab treatment. In the MABEL study, the incidence of IRRs was 2.7 times higher with single premedication compared to combined premedications (H_1_RA plus DEX: 9.6% vs. H_1_RA alone: 25.6%). We hypothesized that the incidence of IRRs with single-drug premedication would be 2.7 times (15.7%) that observed with double-drug premedication in the RAINBOW study. A noninferiority margin (Δ) of + 10% was considered clinically meaningful with reference to the risk ratio of IRR incidence for different cetuximab premedications in the MABEL study. A one-tailed test using a type I error of 0.05 required a sample size of 45 patients in each group to ensure 80% power. *P* values for the noninferiority tests were reported as one-sided, whereas the other *P* values were two-sided. We considered one-sided *P* values of less than 0.025 and two-sided *P* values of less than 0.05 to be statistically significant. All statistical analyses were performed using EZR version 1.61 (Saitama Medical Center, Jichi Medical University, Saitama, Japan), which is a graphical user interface for R (The R Foundation for Statistical Computing, Vienna, Austria). EZR is a modified version of the R commander designed to add statistical functions frequently used in biostatistics [21].

## Results

A total of 99 patients who received RAM plus nab-PTX were identified and reviewed. After applying the inclusion and exclusion criteria, 90 patients were enrolled in two groups: non-H_1_RA (n = 43) and H_1_RA (n = 47). Baseline characteristics and treatment of patients in the non-H_1_RA and H_1_RA groups are shown in Table 1. In the H_1_RA group, first- (diphenhydramine hydrochloride and *d*-chlorpheniramine maleate) and second-generation (bepotastine besylate and fexofenadine hydrochloride) antihistamines accounted for 83% and 17% of the patients, respectively. Allergy history, prior treatment with fluorouracil, and prior treatment with taxanes were significantly higher in patients with non-H_1_RA than in those with H_1_RA (41.9% vs. 21.3%, *P* = 0.042; 95.3% vs. 76.6%, *P* = 0.015; and 41.9% vs. 19.1%, *P* = 0.023, respectively). Although the median RAM dosage was significantly lower in the non-H_1_RA group than in the H_1_RA group (*P* < 0.001), this difference was not clinically significant. The DEX dose was significantly lower in the non-H_1_RA group than in the H_1_RA group (*P* < 0.001). Regarding the infusion time following the second dose of RAM, 30-min infusions occurred more commonly in the non-H_1_RA group compared to the H_1_RA group (Table 2).

**Table 1:** Patient characteristics at ramucirumab initiation

	Non-H_1_RA	H_1_RA	*P* value
	N = 43	N = 47	
Age, years	69 [39–86]	70 [46–86]	0.881
Male	30 (69.8)	36 (76.6)	0.485
ECOG PS			
0	8 (18.6)	7 (14.9)	0.933
1	28 (65.1)	32 (68.1)	
2	6 (14.0)	7 (14.9)	
3	0 (0.0)	1 (2.1)	
Missing	1 (2.3)	0 (0.0)	
History of allergies	18 (41.9)	10 (21.3)	0.042
Foods	6 (14.0)	3 (6.4)	0.301
Drugs	5 (11.6)	(12.8)	1.000
Others	12 (27.9)	2 (4.3)	0.003
RAM dosage, mg/kg	7.7 [4.7–8.4]	7.9 [5.7–8.1]	0.040
RAM infusion rate, mg/min	6.7 [3.7–11.0]	6.7 [4.0–10.5]	0.398
H₁RA			
First generation	–	39 (83.0)	–
Second generation	–	8 (17.0)	–
DEX dosage			
3.3 mg	43 (100.0)	13 (27.7)	< 0.001
4.95 mg	0 (0.0)	1 (2.1)	
6.6 mg	0 (0.0)	33 (70.2)	
Co-administrated aprepitant	2 (4.7)	0 (0.0)	0.225
Treatment line			
1	7 (16.3)	3 (6.4)	0.494
2	23 (53.5)	27 (57.4)	
3	9 (20.9)	10 (21.3)	
4 ≤	4 (9.3)	7 (14.9)	
Previous treatment drugs			
Oxaliplatin	14 (32.3)	19 (40.4)	0.514
Cisplatin	9 (20.9)	10 (21.2)	1.000
Fluoropyrimidine	41 (95.3)	36 (76.6)	0.015
Taxanes	18 (41.9)	9 (19.1)	0.023
Trastuzumab	6 (14.0)	7 (17.0)	1.000
Nivolumab	3 (7.0)	2 (4.3)	0.667
Irinotecan	0 (0.0)	1 (2.1)	1.000
Baseline biological parameters			
WBC, 10^3^/μL	5.1 [2.8–14.1]	5.5 [2.2–16.5]	0.253
AEC, 10^3^/μL	0.08 [0.01–0.42]	0.01 [0–0.70]	0.550
ALC, 10^3^/μL	1.14 [0.65–2.06]	1.29 [0.35–2.84]	0.513

*ECOG* Eastern Cooperative Oncology Group, *PS* performance status, *RAM* ramucirumab, *H*_*1*_*RA* H_1_-receptor antagonists, *DEX* dexamethasone, *WBC* white blood cell count, *AEC* absolute eosinophil count, *ALC* absolute lymphocyte count

Data are presented as interquartile range [IQR] or n (%)

**Table 2:** Dosing characteristics at ramucirumab

	Non-H_1_RA	H_1_RA	*P* value
	N = 271	N = 374	
RAM infusion time (%)			
60 min	150 (55.4)	280 (74.9)	< 0.001
30 min	121 (44.6)	94 (25.1)	
Number of RAM dosing (%)			
1	43 (15.9)	47 (12.6)	0.599
2	30 (11.1)	40 (10.7)	
3	27 (10.0)	34 (9.1)	
4 ≤	171 (63.1)	253 (67.6)	

*RAM* ramucirumab, *H*_*1*_*RA* H_1_-receptor antagonists

**Fig. 1:**
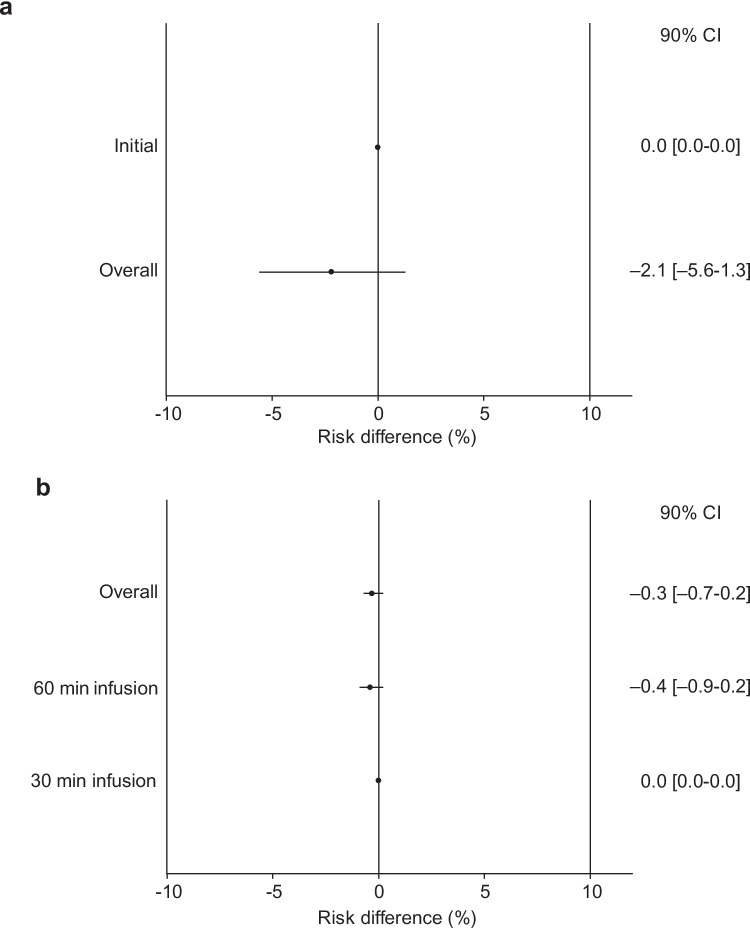
A comparison of the risk difference between non-H_1_RA and H_1_RA is shown with patient (**a**) and dosing (**b**). The solid line indicates a risk difference of 0. The dashed line at a risk difference of 10% indicates the noninferiority margin. *CI* confidence interval

The difference in the incidence of IRRs per patient following RAM plus nab-PTX treatment is shown in Fig. 1A. The primary endpoint, defined as the incidence of IRRs at the initial RAM infusion, was not observed in either group. The proportion of patients who developed IRRs during the overall period was 0% for non-H_1_RA and 2.1% for H_1_RA, with a risk difference of –2.1% (90% confidence interval: –5.6%–1.3%). IRRs in 30-min infusion duration were not observed in either group (Fig. 1B). Thus, the noninferiority of non-H_1_RA to H_1_RA in the incidence of RAM-induced IRRs was demonstrated.

Only one patient in the H_1_RA group exhibited drowsiness, which was suspected to be a result of premedication, as indicated by the electronic medical records.

## Discussion

This study aimed to determine whether premedication of H_1_RA for RAM-induced IRRs could be omitted in patients with gastric cancer receiving RAM plus nab-PTX. Indeed, we demonstrated the noninferiority of premedication for non-H_1_RA compared to that for H_1_RA.

In previous studies, risk factors for RAM-induced IRRs included living in Asia, receiving chemotherapy, age, and no premedication [22, 23]. Goto et al. demonstrated that IRRs were not observed in patients receiving H_1_RA-free RAM regimens for solid cancers [19]. Another study found no incidence of IRRs without premedication of H_1_RA in RAM plus levofolinate, fluorouracil, and irinotecan (FOLFIRI) for colorectal cancer [24]. Our study evaluating RAM plus nab-PTX for gastric cancer showed results similar to those of previous studies. To our knowledge, this is the first study to demonstrate the noninferiority of premedication for H_1_RA omission compared to H_1_RA in RAM-induced IRRs receiving RAM plus nab-PTX for gastric cancer. If RAM in combination with nab-PTX could be administered without H_1_RA, it would facilitate outpatient chemotherapy, alleviating concerns regarding the side effects associated with H_1_RA.

RAM plus nab-PTX, which is a low emetogenic risk regimen, is recommended as prophylaxis along with DEX in the current guidelines [16]. The results of our study suggest that premedication with DEX in RAM plus nab-PTX may be useful for the prevention of IRRs, nausea, and vomiting. In the MABEL study, the frequency of IRRs was reduced when corticosteroids were administered in addition to H_1_RA as a premedication for cetuximab in colorectal cancer [20]. However, unlike cetuximab, RAM is a fully human monoclonal antibody, and the incidence of IRRs is lower than that with cetuximab (6.6% vs. 15%) [7, 25]. Therefore, DEX premedication alone may be sufficient to prevent IRRs with RAM.

The antihistamines used in the H_1_RA group consisted of both first-generation and second-generation antihistamines. A study comparing a second-generation antihistamine, cetirizine, with a first-generation antihistamine, diphenhydramine, in preventing chemotherapy-associated hypersensitivity reactions (PTX, cetuximab, rituximab) found similar rates of IRRs [26]. Thus, different generations of antihistamines may not significantly affect the occurrence of IRRs.

H_1_RA, which has anticholinergic properties, is less likely to be used in patients with angle-closure glaucoma or benign prostatic hyperplasia. Adverse side effects of H_1_RA include drowsiness and dizziness, which can cause impaired driving [15]. The omission of H_1_RA from outpatient chemotherapy is beneficial for patients who drive cars. The current results represent potentially significant findings that could enhance the quality of life for patients undergoing outpatient chemotherapy.

This study had some limitations. First, despite the multicenter collaboration in this study, the frequency of RAM-induced IRRs was low, and the sample size for finding IRRs was small. The incidence of IRRs with H_1_RA was lower than that reported in the RAINBOW study, which was used as a reference to set the sample size. The only adverse side effect assessed using H_1_RA was drowsiness, which was difficult to measure in many cases where there were no detailed descriptions in the electronic medical records. The retrospective nature of this study may have led to the under-reporting of IRRs and drowsiness. Second, this retrospective study included patients from diverse backgrounds across the two groups, leading to variations among centers. These variations contributed to the differences in allergy history, DEX doses, and types of prior treatment drugs between the groups. We conducted subgroup analyses based on patient characteristics and risk differences according to allergy history and a DEX dose of 3.3 mg. The results of these analyses, provided in the supplementary file, confirmed our findings. In addition, there was a difference in the infusion time of RAM in the dosing characteristics between the two groups. It cannot be overlooked that disparities between centers might have impacted the outcomes. However, the previously reported risk factors for IRRs, such as living in Asia, receiving chemotherapy, and age, did not differ between the two groups. Reducing the infusion time of RAM from 60 to 30 min was reported to not affect IRRs [22, 23]. In this study, the non-H_1_RA group received a lower dose of DEX, and no IRRs occurred; however, this is unlikely to be the underlying reason for the relative inferiority of the non-H_1_RA group compared to the H_1_RA group. This study, which involved premedication with DEX, has not clarified whether H_1_RA can be safely omitted in the context of RAM plus nab-PTX without the concurrent use of DEX. The absence of premedication poses a known risk factor for IRRs [22, 23], suggesting that the likelihood of RAM-induced IRRs may increase during H_1_RA omission, particularly when DEX is replaced with antiemetic medications targeting the 5-hydroxytryptamine-3 receptor, such as granisetron. These limitations cannot be overlooked, and further extensive prospective studies are imperative to comprehensively understand these outcomes.

## Conclusion

In conclusion, the results of this study suggest that RAM plus nab-PTX can be administered without H_1_RA if premedicated with DEX. RAM plus nab-PTX may be a more favorable chemotherapeutic option than RAM plus PTX, which requires H_1_RA in patients with gastric cancer.

## Supplementary Information

Below is the link to the electronic supplementary material.

Supplementary file1 (PDF 164 KB)

## Data Availability

No datasets were generated or analysed during the current study.
