# Supplementary material for: Dexamethasone is non-inferior to antihistamine plus dexamethasone premedication in preventing ramucirumab plus nab-paclitaxel infusion-related reactions in gastric cancer: a multicenter retrospective study
Source: Support Care Cancer. 2024 Oct 7;32(10):704. doi: 10.1007/s00520-024-08910-8 (PMC11456548; doi:10.1007/s00520-024-08910-8)
Supplement: Supplementary file 1 — Supplementary file1 (PDF 164 KB) [file 520_2024_8910_MOESM1_ESM.pdf]

Dexamethasone is non-inferior to antihistamine plus dexamethasone premedication in preventing ramucirumab plus nab-paclitaxel infusion-related reactions in gastric cancer: a multicenter retrospective study

Supportive Care in Cancer

Yutaka Negoro[1]*, Taichi Maeda[2], Hiroyuki Igarashi[3], Mina Shigemori[1], Toshihiro Tanaka[1], Yukio Ito[3], Norihiko Tanizawa[2], Shota Nishikawa[2], Jyunya Ogawa[2], Yukio Kamitani[1], Kyohei Watanabe[4], Hitoshi Tsukamoto[1], Nobuyuki Goto[1]

Affiliations
[1]Department of Pharmacy, University of Fukui Hospital, Yoshida-gun, Fukui, Japan
[2]Department of Pharmacy, Japanese Red Cross Fukui Hospital, Fukui, Japan
[3]Department of Pharmacy, Fukui-ken Saiseikai Hospital, Fukui, Japan
[4]Medical Research Support Center, University of Fukui Hospital, Yoshida-gun, Fukui, Japan

Corresponding author
Yutaka Negoro
Email: ynegoro@u-fukui.ac.jp

Supplemental Table 1 ─ Patient characteristics at ramucirumab initiation in history of allergies

	Non-H_1RA N = 18	H_1RA N = 10	*P* value
Age, years	68 [46–77]	62 [46–86]	0.943
Male	11 (61.1)	5 (50.0)	0.698
ECOG PS			
0	2 (11.1)	1 (10.0)	0.891
1	12 (66.7)	6 (60.0)	
2	3 (16.7)	3 (30.0)	
Missing	1 (2.3)	0 (0.0)	
RAM dosage, mg/kg	7.8 [5.8–8.4]	7.9 [7.4–8.0]	0.397
RAM infusion rate, mg/min	5.9 [4.8–9.5]	6.3 [4.2–8.5]	0.962
DEX dosage			
3.3mg	18 (100.0)	4 (40.0)	0.001
6.6mg	0 (0.0)	6 (60.0)	
Co-administrated aprepitant	1 (5.6)	0 (0.0)	1.000
Treatment line			
1	4 (22.2)	0 (0.0)	0.270
2	8 (44.4)	8 (80.0)	
3	4 (22.2)	1 (10.0)	
4 ≤	2 (11.1)	1 (10.0)	
Previous treatment drugs			
Oxaliplatin	6 (33.3)	6 (60.0)	0.243
Cisplatin	2 (11.1)	3 (30.0)	0.315
Fluoropyrimidine	13 (72.2)	9 (90.0)	0.375
Taxanes	7 (38.9)	2 (20.0)	0.417
Trastuzumab	1 (5.6)	1 (10.0)	1.000
Nivolumab	2 (11.1)	0 (0.0)	0.524
Baseline biological parameters			
WBC, $10^3/\mu L$	4.8 [2.8–14.1]	5.8 [3.1–11.3]	0.093
AEC, $10^3/\mu L$	0.06 [0.02–0.41]	0.01 [0–0.70]	0.829
ALC, $10^3/\mu L$	1.14 [0.65–1.98]	1.24 [0.59–2.38]	0.737

ECOG Eastern Cooperative Oncology Group, *PS* performance status, *RAM* ramucirumab, *H_1RA* H_1-receptor antagonists, *DEX* dexamethasone, *WBC* white blood cell count, *AEC* absolute eosinophil count, *ALC* absolute lymphocyte count

Data are presented as interquartile range [IQR] or n (%)

Supplemental Table 2 ─ Patient characteristics at ramucirumab initiation in Non-history of allergies

	Non-H_1RA N = 25	H_1RA N = 37	*P* value
Age, years	70 [39–86]	70 [48–85]	0.863
Male	19 (76.0)	31 (83.8)	0.521
ECOG PS			
0	6 (24.0)	6 (16.2)	0.871
1	16 (64.0)	26 (70.3)	
2	3 (12.0)	4 (10.8)	
3	0 (0.0)	1 (2.7)	
RAM dosage, mg/kg	7.7 [4.7–8.2]	7.9 [5.7–8.1]	0.039
RAM infusion rate, mg/min	6.7 [3.7–11.0]	6.7 [4.0–10.5]	0.560
DEX dosage			
3.3mg	25 (100.0)	9 (24.3)	<0.001
4.95mg	0 (0.0)	1 (2.7)	
6.6mg	0 (0.0)	27 (73.0)	
Co-administrated aprepitant	1 (4.0)	0 (0.0)	0.403
Treatment line			
1	3 (12.0)	3 (8.1)	0.775
2	15 (60.0)	19 (51.4)	
3	5 (20.0)	9 (24.3)	
4 ≤	2 (8.0)	6 (16.2)	
Previous treatment drugs			
Oxaliplatin	8 (32.0)	17 (45.9)	0.304
Cisplatin	7 (28.0)	7 (18.9)	0.538
Fluoropyrimidine	21 (84.0)	27 (73.0)	0.367
Taxanes	11 (44.0)	7 (18.9)	0.047
Trastuzumab	5 (20.0)	7 (18.9)	1.000
Nivolumab	1 (4.0)	2 (5.4)	1.000
Baseline biological parameters			
WBC, 10^3/μL	5.4 [2.9–10.4]	5.4 [2.2–16.5]	0.943
AEC, 10^3/μL	0.09 [0.07–0.42]	0.11 [0–0.43]	0.994
ALC, 10^3/μL	1.14 [0.74–2.06]	1.29 [0.35–2.84]	0.631

ECOG Eastern Cooperative Oncology Group, *PS* performance status, *RAM* ramucirumab, *H_1RA* H_1-receptor antagonists, *DEX* dexamethasone, *WBC* white blood cell count, *AEC* absolute eosinophil count, *ALC* absolute lymphocyte count

Data are presented as interquartile range [IQR] or n (%)

Supplemental Table 3 ― Patient characteristics at ramucirumab initiation in DEX dosage of 3.3mg group

	Non-H_1RA N = 43	H_1RA N = 13	*P* value
Age, years	69 [39–86]	63 [49–83]	0.294
Male	30 (69.8)	11 (84.9)	0.477
ECOG PS			
0	8 (18.6)	3 (23.1)	0.450
1	28 (65.1)	6 (46.2)	
2	6 (14.0)	4 (30.8)	
Missing	1 (2.3)	0 (0.0)	
History of allergies	18 (41.9)	4 (30.8)	0.535
Foods	6 (14.0)	1 (7.7)	1.000
Drugs	5 (11.6)	1 (7.7)	0.480
Others	12 (27.9)	2 (15.4)	0.003
RAM dosage, mg/kg	7.7 [4.7–8.4]	8.0 [7.9–8.0]	<0.001
RAM infusion rate, mg/min	6.7 [3.7–11.0]	6.7 [5.3–10.5]	0.490
Co-administrated aprepitant	2 (4.7)	0 (0.0)	1.000
Treatment line			
1	7 (16.3)	0 (0.0)	0.257
2	23 (53.5)	10 (76.9)	
3	9 (20.9)	1 (7.7)	
4 ≤	4 (9.3)	2 (15.4)	
Previous treatment drugs			
Oxaliplatin	14 (32.3)	6 (46.2)	0.510
Cisplatin	9 (20.9)	3 (23.1)	1.000
Fluoropyrimidine	41 (95.3)	11 (84.6)	0.227
Taxanes	18 (41.9)	2 (15.4)	0.106
Trastuzumab	6 (14.0)	3 (23.1)	0.419
Nivolumab	3 (7.0)	1 (7.7)	1.000
Irinotecan	0 (0.0)	1 (7.7)	0.232
Baseline biological parameters			
WBC, $10^3/\mu L$	5.1 [2.8–14.1]	4.6 [2.2–9.5]	0.336
AEC, $10^3/\mu L$	0.08 [0.01–0.42]	0.09 [2–0.70]	0.741
ALC, $10^3/\mu L$	1.14 [0.65–2.06]	1.26 [0.35–2.38]	0.884

ECOG Eastern Cooperative Oncology Group, *PS* performance status, *RAM* ramucirumab, H_1RA H_1-receptor antagonists, *DEX* dexamethasone, *WBC* white blood cell count, *AEC* absolute eosinophil count, *ALC* absolute lymphocyte count

Data are presented as interquartile range [IQR] or n (%)

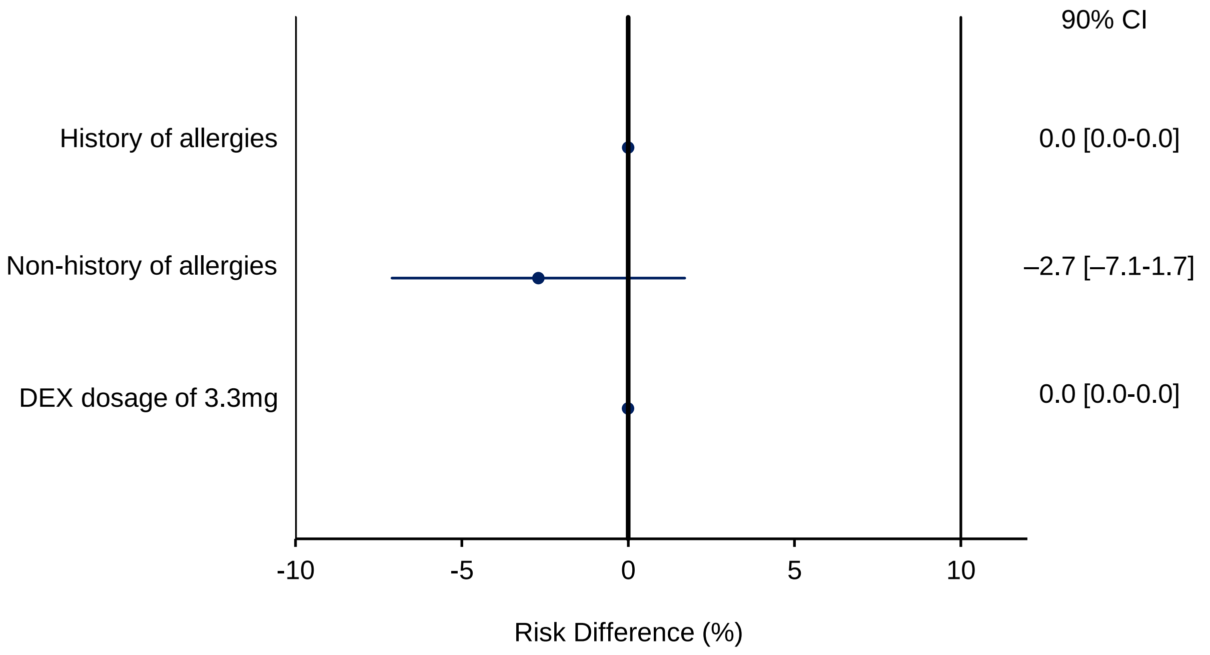

Supplemental Figure 1 ─ A comparison of the risk difference between non-H_1RA and H_1RA is shown with history of allergies, non-history of allergies and DEX dosage of 3.3mg patients. The solid line indicates a risk difference of 0. The dashed line at a risk difference of 10% indicates the non-inferiority margin. *CI* confidence interval, *DEX* dexamethasone
